# A systematic review and meta-analysis of ambient temperature and precipitation with infections from five food-borne bacterial pathogens

**DOI:** 10.1017/S0950268824000839

**Published:** 2024-08-22

**Authors:** Naveen Manchal, Megan K. Young, Maria Eugenia Castellanos, Peter Leggat, Oyelola Adegboye

**Affiliations:** 1 Public Health and Tropical Medicine, College of Public Health, Medical and Veterinary Sciences, James Cook University, Townsville, QLD, Australia; 2 Metro North Public Health Unit, Metro North Hospital and Health Service, Brisbane, Australia; 3 School of Medicine and Dentistry, Griffith University, Gold Coast, Australia; 4 Faculty of Medicine, School of Public Health, University of Queensland, Brisbane, QLD, Australia; 5 Australian Institute of Tropical Health and Medicine, James Cook University, Townsville, QLD, Australia; 6 World Health Organization Collaborating Centre for Vector-Borne and Neglected Tropical Diseases, James Cook University, Townsville, QLD, Australia; 7 School of Public Health, Faculty of Health Sciences, University of the Witwatersrand, Johannesburg, South Africa; 8 Menzies School of Health Research, Charles Darwin University, Darwin, NT, Australia

**Keywords:** Gastroenteritis, bacteraemia, temperature, precipitation, infectious disease

## Abstract

Studies on climate variables and food pathogens are either pathogen- or region-specific, necessitating a consolidated view on the subject. This study aims to systematically review all studies on the association of ambient temperature and precipitation on the incidence of gastroenteritis and bacteraemia from *Salmonella*, *Shigella*, *Campylobacter*, *Vibrio*, and *Listeria* species. PubMed, Ovid MEDLINE, Scopus, and Web of Science databases were searched up to 9 March 2023. We screened 3,204 articles for eligibility and included 83 studies in the review and three in the meta-analysis. Except for one study on *Campylobacter*, all showed a positive association between temperature and *Salmonella, Shigella, Vibrio sp.*, and *Campylobacter* gastroenteritis. Similarly, most of the included studies showed that precipitation was positively associated with these conditions. These positive associations were found regardless of the effect measure chosen. The pooled incidence rate ratio (IRR) for the three studies that included bacteraemia from *Campylobacter* and *Salmonella sp.* was 1.05 (95 per cent confidence interval (95% CI): 1.03, 1.06) for extreme temperature and 1.09 (95% CI: 0.99, 1.19) for extreme precipitation. If current climate trends continue, our findings suggest these pathogens would increase patient morbidity, the need for hospitalization, and prolonged antibiotic courses.

## Introduction

Worldwide, 33 million years of healthy lives are lost each year to food-borne illness, which is underestimated [1]. Studies have shown that warmer climates and heat waves increase the incidence of Salmonellosis and Campylobacteriosis [2, 3]. However, different climate variables can affect each food-borne pathogen differently. The association between temperature rise and increased incidence of infection is more consistent with salmonellosis than with *Listeria* infection [4]. A meta-analysis showed the pooled relative risk (RR) for each 1-degree rise in temperature for salmonellosis was 1.05 (95% confidence interval (C):1.04–1.07) [5]. For *Vibrio* infections, an increase in water (not air) temperature is associated with an increased incidence of infection [4]. The intensity and rapidity of exposure to the climate variable also determine the risk of infection.

In addition to the number of infections, it is also important to study the severity of the disease. Are these infections limited to gastroenteritis, or is there a trend for more invasive infections like bacteraemia? The impact of bacteraemia compared to gastroenteritis is greater, with increased morbidity and mortality, hospitalization, and health services costs [6, 7]. With climate change and more difficult conditions for environmental pathogens, bacteraemia may reflect increased virulence from these organisms. A 10-year analysis of passive surveillance data in Queensland, Australia, noted a rise in the incidence of invasive salmonellosis, particularly in the elderly [8]. Ninety-two per cent of these invasive infections were diagnosed on blood culture [8]. *Salmonella Virchow* was the most common species identified [8]. The contributory factors for the increased invasiveness of these infections were unclear. It has been projected that compared to the years of life lost to disabilities (YLD) in 2000, salmonellosis would contribute to a 9–48% increase in YLD by 2030 due to temperature changes from climate change [9].

The relationship between climate change and food/waterborne disease is complex. There are temporal and regional variations across the world affected by behavioural changes in populations that increase the risk of these illnesses. While cholera, enteric fever, and bacillary dysentery predominate in the Indian subcontinent and Africa, non-cholera *Vibrio* species and non-typhoidal *Salmonella* and *Campylobacter* infections are prevalent in the temperate regions of the world. There is heterogeneity in the studies reporting an association between climate variables and enteric pathogens, with varied methodologies and modelling strategies.

Existing literature on the impact of climate variables on food-borne pathogens has been restricted to a particular variable [5] or pathogen [10]. We formulated the research question to determine the effect of ambient temperature (including heat waves) and precipitation (including floods) on the incidence of pathogen-specific infections – gastroenteritis and bacteraemia.

## Methods

### Search strategy

This systematic review followed the Preferred Reporting Items for Systematic Reviews and Meta-Analyses (PRISMA) guidelines. Searches for published literature in English on the impact of climate change on infections from *Salmonella*, *Shigella, Campylobacter, Vibrio*, and *Listeria* infections were conducted. MEDLINE (Ovid), Scopus, PubMed, and Web of Science electronic databases were searched without any restrictions on date range.

### Inclusion and exclusion criteria

All published articles on the pathogens of interest and one or more climate variables were eligible for inclusion. No time frame was applied as the effect of climate variables on bacteria has not been a recent development. We excluded studies not in English and those not on selected pathogens or climate variables of interest and review articles. Conference abstracts and posters were not included.

### Data extraction

One author (NM) screened the abstracts, shortlisted the studies for full-text assessment, and determined inclusion in the review upon examination of the full text. The final list of eligible studies for meta-analysis was checked by two authors (NM and OA). The studies were tabulated by the pathogen of study and data on publication year, study location, study time period, number of cases, population number, climate variable exposure, exposure lag, quantitative estimation of risk, modelling strategy, and key findings, and reported statistics of adjusted analyses were extracted into a purpose-built database. The risk estimates that the studies reported were the correlation coefficient (r), RR, odds ratio (OR), and incidence rate ratio (IRR).

### Quality appraisal

We used the ROBINS-E tool as a guide for assessing the risk of bias within the included studies [11]. The tool is validated for use in non-randomized ecological studies. The tool consists of seven domains: bias confounding, exposure and outcome measurements, participation selection, post-exposure intervention, missing data, and reporting bias. Each domain is assessed through signalling questions to make judgements on the risk of bias in the domain, the predicted direction of bias, and whether the risk of bias threatens conclusions regarding the exposure having an effect on the outcome. If the risk of bias was considered ‘high enough to change the direction of the outcomes’, the domain was marked as high risk. If the bias was ‘very low’, the domain was marked as low risk. Studies were considered high quality if the overall judgement suggested a low risk of bias in at most one domain. If there were ‘some concerns of bias’ in at least two domains, they were considered moderate in quality, and if there were three or more domains with ‘high or very high risk of bias’, they were low in quality.

### Meta-analysis

Studies that had included cases of bacteraemia were shortlisted for meta-analysis. We used random-effects models with inverse-variance weighting to pool the IRR estimates for each pathogen together with their 95% confidence interval (CI). The between-study heterogeneity was evaluated using *I*^2^ statistics as the proportion of variability in effect estimates that is not attributed to sampling error. Following Higgins et al. 2019 [12], a threshold of *p* < 0.1 was used to indicate statistical significance, and *I*^2^ values of 25%, 50%, and 75% were considered to represent low, moderate, and considerable heterogeneity, respectively. The statistical analysis was carried out in R version 4.2.2 [13] package meta and metaphor [14].

## Results

### Characteristics of included studies

**Figure 1.**
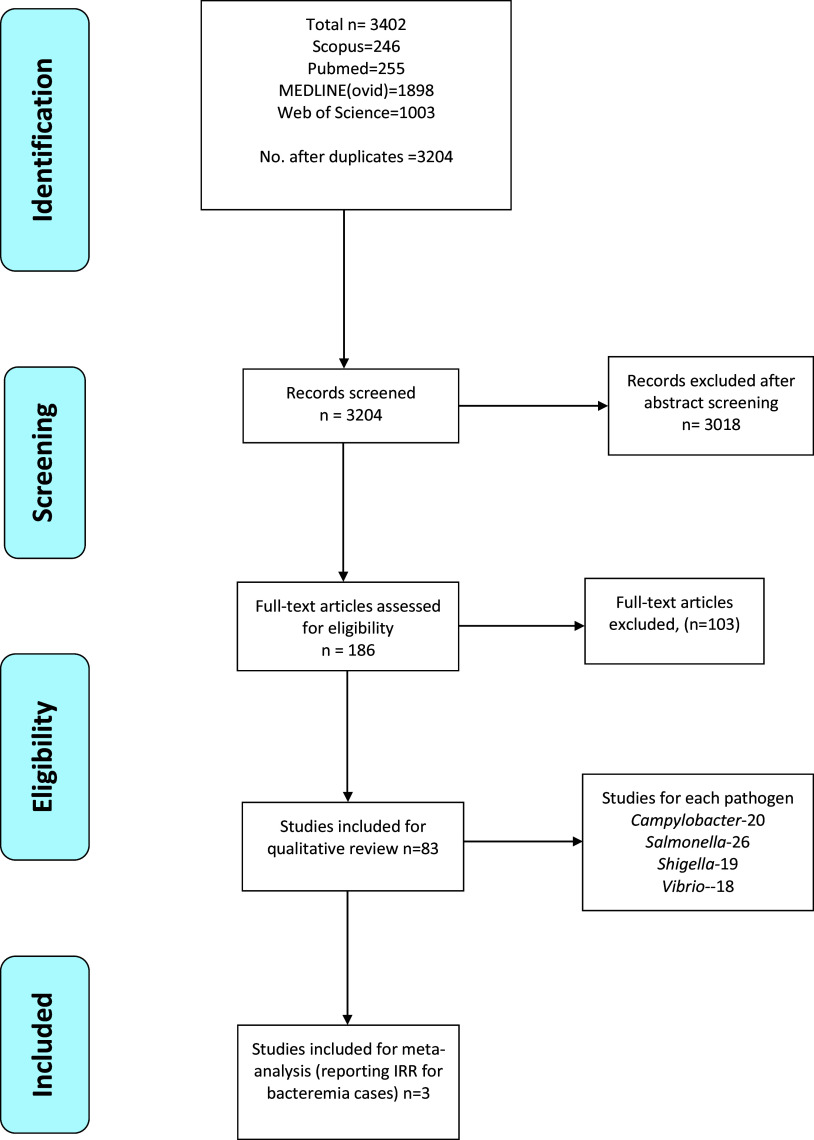
PRISMA flow chart showing the study selection process.

A total of 3,402 studies were obtained from the databases, and after sorting duplicates, 3,204 abstracts were screened. Out of the 186 articles shortlisted for full-text reading and eligibility, 83 were included in the qualitative review, and three were chosen for meta-analysis (Figure 1). Publication years ranged from 2007 to 2019 (the year of data extraction), with the great majority of included articles (n = 69; 73%) published since 2015 (Figure 1). The grouping of studies by countries and pathogens is summarized in Table 1.

**Table 1.** Grouping of studies by regions of study, pathogens, and main findings with climate variable associations

Region of study (number)	Time period of studies	Pathogen	Infections reported	Association with rise in ambient temperature	Association with rise in precipitation
Europe (30), UK (9), Germany (6), Italy (4), Sweden (4), Denmark (2), France (2), Georgia (1), Finland (1)	1981–2015	*Campylobacter* *Salmonella* *Vibrio sp.*	Gastroenteritis and bacteraemia Gastroenteritis Gastroenteritis, wound infections	Positive associations Positive association in all studies Positive association with sea temperature	6 out of 9 studies reported a positive association No association in temperate regions
Australia (24)	1990–2019	*Campylobacter* *Salmonella*	Gastroenteritis Gastroenteritis	Positive association in Brisbane not Adelaide Positive association in all studies	No association
Asia (21), China (11), Iran (4), India (1), Jordan (1), Korea (1), Nepal (1), Philippines (1), Taiwan (1)	1984–2018	*Salmonella* *Shigella* *Vibrio sp.*	Gastroenteritis Gastroenteritis Gastroenteritis	Positive association in all studies Positive association in all studies Positive association	1990 studies out of 16 reported positive association 9 out of 10 studies found positive association with floods 8 out of 9 studies had a positive association
North and South America (17), USA (15), Canada (1), Brazil (1)	1992–2018	*Campylobacter* *Salmonella* *Shigella* *Vibrio parahaemolyticus*	Gastroenteritis and bacteraemia Gastroenteritis and bacteraemia Gastroenteritis Gastroenteritis	Positive association Positive association Positive association	Positive in one study Positive association for extreme precipitation Positive
Africa (3), Ethiopia (1), Ghana (1)	2002–2008	*Vibrio*	Gastroenteritis	Positive association	Positive association

All studies in the qualitative review are tabulated. Twenty studies for *Campylobacter* and twenty-six, nineteen, and eighteen studies for *Salmonella, Shigella*, and *Vibrio* species, respectively, were identified. The maximum lagged week was 52 weeks for *Vibrio sp.* and 9, 12, and 4 weeks for *Campylobacter, Salmonella*, and *Shigella* species, respectively. The majority of the articles were scored as having some concerns for bias in at least two domains and were categorized as moderate in quality in the overall judgement (Table S1 in the Supplementary Material).

Ten high-quality studies were identified for *Campylobacter*, and 11 were of moderate quality. Out of these, three had cases that included bacteraemia [15–17]. Twelve studies on Salmonellosis were high quality. Four studies included patients with bacteraemia [10, 18–20]. With shigellosis, none of the studies specifically discussed bacteraemia and three studies were of high quality. With *Vibrio sp.*, four studies were of high quality.

### Overview of pathogens and effect of temperature and precipitation

#### Campylobacter species

**Figure 2.**
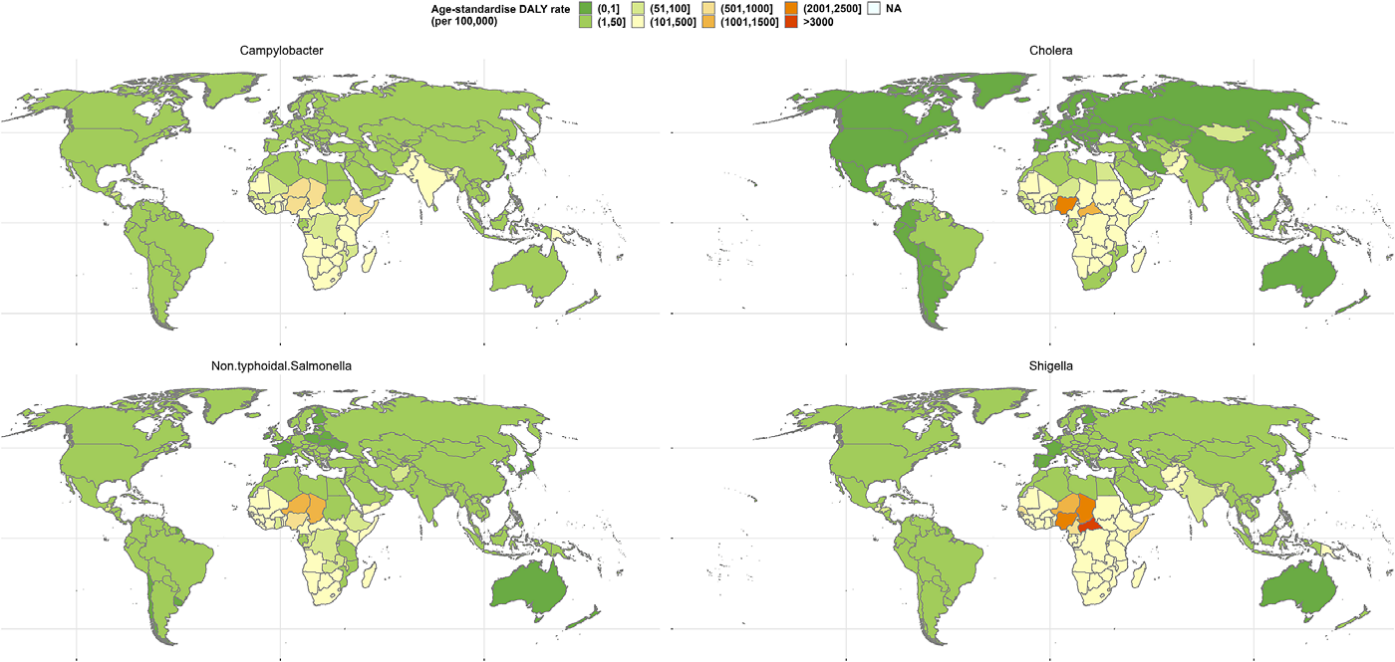
Global distribution of the burden of Campylobacter, cholera, non-typhoid Salmonella, and Shigella. Source: GBD Results tool: Global Burden of Disease Collaborative Network. Global Burden of Disease Study 2019 (GBD 2019) Results, Seattle, United States: Institute for Health Metrics and Evaluation (IHME), 2020.

**Figure 3.**
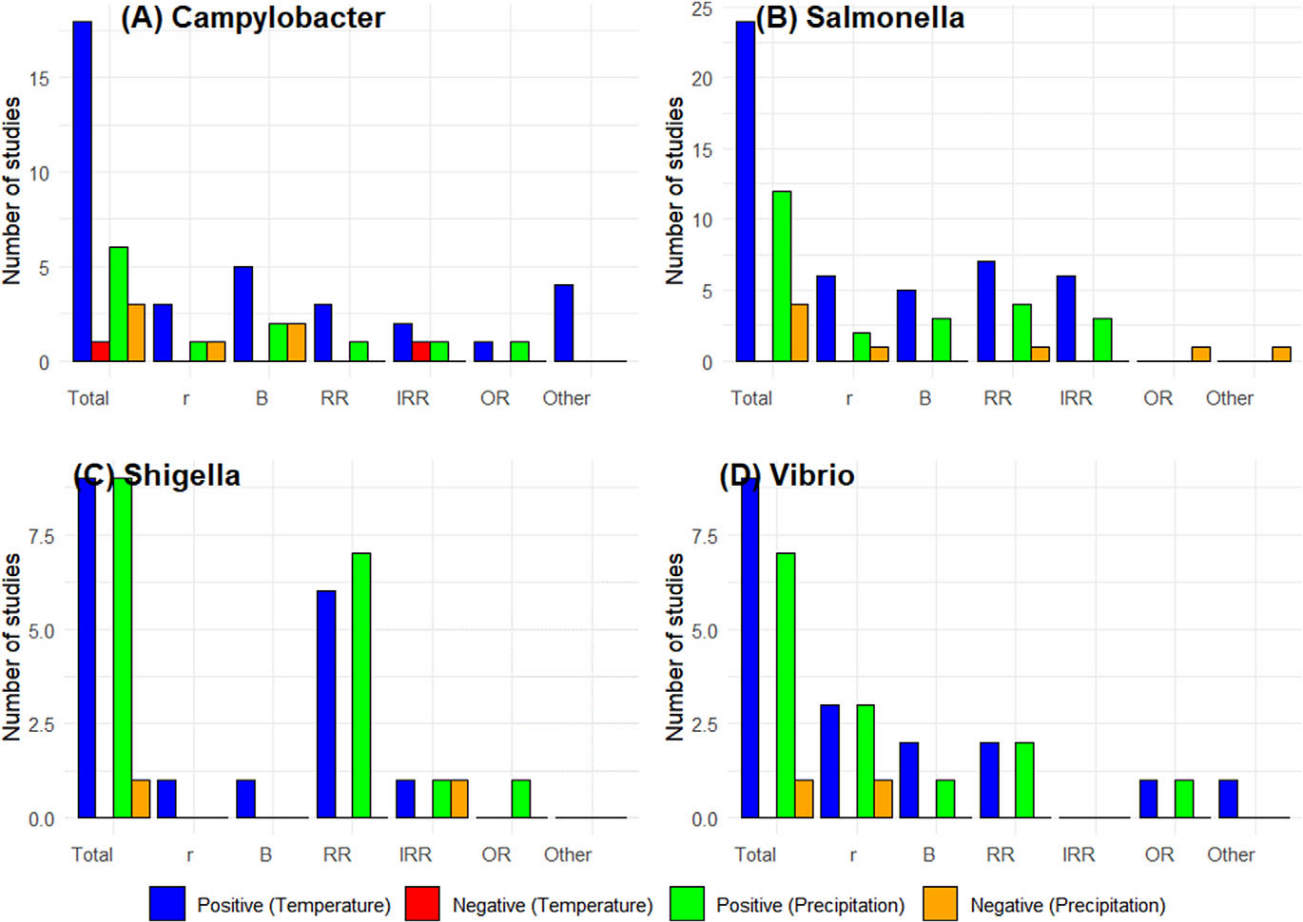
Graphs summarizing the estimated effects (r, beta, RR, IRR, and OR) of temperature and precipitation on specific pathogens. (a) Campylobacter, (b) Salmonella, (c) Shigella, and (d) Vibrio.

The burden of Campylobacteriosis is high in the Americas and Europe, predominantly in the temperate regions (Figure 2), with the United States of America (USA) and United Kingdom (UK) reporting age-standardized disability-adjusted life year (DALY) of 7.55 and 9.4, respectively [21]. Studies on Campylobacteriosis were predominantly conducted in Europe, North America, and Oceania (Table 1). Campylobacteriosis had a positive association with ambient temperature, whether it was measured as a weekly maximum, monthly, or daily average and extreme heat (Table 2 and Figure 3a). This was true not only for gastroenteritis but also for bacteraemia. The rise in cases was mostly found in a temperature range between 10 and 25 ° C. Out of the 20 studies, 19 (95%) reported a positive association with temperature. With precipitation, six out of nine studies described a positive association (Figure 3a and Table 3).

**Table 2.** Studies on *Campylobacter* with temperature as the climate variable, stratified by type of temperature measurement

Number	Study	Region	Time period	Infection studied	Case number	Population number	Analytical method	Key findings and reported statistic^a^
***Weekly maximum temperature***								
1	Bi 2008 [22]	Brisbane, Australia	1990–2005	Weekly lab–confirmed gastroenteritis	14,697	1,600,000	Time–series Poisson regression	R = 0.01 at lag 6 weeks
2	Bi 2008 [22]	Adelaide, Australia	1990–2006	Weekly lab–confirmed gastroenteritis	20,211	n/a	Time–series Poisson regression	B = 0.01 at lag 9 weeks
3	Kovats 2005 [24]	Czech Republic, England and Wales, Scotland, Spain, Switzerland, Denmark	1991–2002	Weekly lab–confirmed infections	75,312	n/a	Logistic regression	Maximum OR 1.3 after lag 14 weeks Temperature range (TR) 10–30 °C
***Weekly mean temperature***								
4	Fleury et al. 2006 [26]	Alberta, Canada	1992–2000	Weekly lab–confirmed infections	1743	2,696,826	Generalized linear model	RR = 1.025 (1.02, 1.03) at lag 3 weeks TR 0–20 °C
5	Lake et al. 2009 [35]	UK	1989–2006	Weekly lab–confirmed infections	n/a	n/a	Regression analysis	RR = 1.0534 (1.03, 1.08) at lag 0 week. TR 10–20^0^ C
6	Kuhn 2020 [3]	Nordic countries (2000–2015)	2000–2015	Weekly mean temperature, heat wave gastroenteritis and bacteraemia	64,034	26,000,000	Poisson regression	B = 0.09 for temperature and − 0.1 for heat wave TR −35.3–32.8 °C
7	Patrick et al. 2004 [73]	Denmark	1998–2001	Weekly lab–confirmed infections	16,305	4,400,000	Linear regression	Max. temperature 4 weeks prior had 68% variance TR 13–20 °C
8	Rosenberg et al. 2018 [74]	Israel	1999–2010	Weekly lab–confirmed gastroenteritis	29,762	n/a	Poisson generalized additive	1–deg. rise associated with 16.1% increase in *Campylobacter jejuni* and 18.8% increase in *Campylobacter coli* TR 15–30 °C
9	Tam et al. 2006 [77]	UK	1989–1999	Weekly lab–confirmed gastroenteritis	623,817	n/a	Negative binomial regression	RR = 1.05 (1.03, 1.06) at lag 6 weeks up to a threshold of 14 deg. TR 0–14^0^ C
10	White et al. 2009 [80]	Philadelphia, USA	1994–2007	Weekly lab–confirmed gastroenteritis	1,477	1,517,550	Poisson regression	IRR = 1.041, warm humid weather increases risk
11	Yun et al. 2016 [81]	Germany	2004–2007	Weekly clinical and lab–confirmed cases	n/a	n/a	Regression analysis	Positive correlation at lag 5 weeks TR 10–25 °C
***Monthly mean temperature***								
12	Kim et al. 2015 [94]	South Korea	2003–2012	Monthly temperature and outbreaks of gastroenteritis	n/a	n/a	Pearson correlation	r = 0.66 Incidence calculated by dividing pathogen–specific outbreak by total food–borne outbreaks
13	Carev et al. 2018 [23]	Croatia	2007–2012	Monthly counts of lab–confirmed infections	2,658	454,798	Linear regression	r = 0.58 TR 10–25 °C
14	Vucković et al. 2011 [76]	Croatia	2003–2007	Annual counts of gastroenteritis	1,242	305,505	Multiple regression	B = 0.83 in 2003 TR 10–25^0^ C
15	Sanderson et al. 2018 [75]	UK	2004–2009	Monthly lab–confirmed infections	n/a	n/a	Autoregressive moving average	B = 0.07 at lag 4 weeks
***Extreme heat and daily temperature***								
16	Soneja et al. 2016 [16]	USA	2002–2012	Extreme heat and gastroenteritis and bacteraemia	4,804	5,900,000	Multivariate binomial regression	IRR =1.04 ETT_95_
17	Djennad et al. 2019 [72]	UK	2005–2009	Weekly lab–confirmed gastroenteritis, daily mean temperature	n/a	n/a	Generalized time series	B = 7.32 accounting for 33.3.% of cases at lag 2 weeks TR 10–25 °C
18	Milazzo et al. 2017 [78]	Adelaide, Australia	1990–2012	Lab–confirmed infections	35,601	n/a	Poisson regression model	IRR = 0.906 with heat waves, no effect of temperature in warm season, and no lag effect
19	Spencer et al. 2012 [79]	New Zealand	2001–2007	Lab–confirmed infections	n/a	n/a	Poisson regression model	Spatial and temporal risk factors studied and no temporal risk factors identified

a r = correlation coefficient, B = beta coefficient, RR = relative risk, OR = odds ratio, IRR = incidence rate ratio, n/a = not available.

**Table 3.** Studies on *Campylobacter* with precipitation as the climate variable, stratified by type of precipitation measurement

Number	Study on organism	Region	Time period	Infection studied	Case number	Population number	Analytical method	Key findings and reported statistic^a^
***Weekly total precipitation***								
1	Bi 2008 [22]	Adelaide, Australia	1990–2006	Weekly lab–confirmed gastroenteritis	20,211	n/a	Time–series Poisson regression	B = 0.01 at lag 1 week, r = 0.05
2	Kuhn et al. 2020 [15]	Nordic countries (2000–2015)	2000–2015	Gastroenteritis and bacteraemia	64,034	2,600,0000	Poisson regression	B = 0.3 Precipitation range (PR) 0–105 mm
3	Patrick et al. 2004 [73]	Denmark	1998–2001	Weekly lab–confirmed infections	16,305	4,400,000	Linear regression	r = 0.06 at lag 4 weeks
4	Djennad et al. 2019 [72]	UK	2005–2009	Weekly lab–confirmed gastroenteritis	n/a	n/a	Generalized time series	B = 9.36 at lag 1 week PR 0–80 mm
***Monthly mean precipitation***								
5	Carev et al. 2018 [23]	Croatia	2007–2012	Monthly counts of lab–confirmed infections	2,658	454,798	Linear regression	r = 0.04 PR 0–25 mm
6	Sanderson et al. 2018 [75]	UK (2004–2009)	2004–2009	Monthly lab–confirmed infections	n/a	n/a	Autoregressive moving average	B = 0.01 at lag 4 weeks
***Daily and extreme precipitation***								
7	Nichols et al. 2009 [25]	England	1910–1999	Lab–confirmed outbreaks (2 or more cases) and daily rainfall	n/a	n/a	Conditional logistic regression	OR 2.88 2 weeks prior to outbreak with rainfall>40 mm. (Outbreaks vs. control years were 7 vs. 2)
8	Soneja et al. 2016 [16]	USA	2002–2012	Gastroenteritis and bacteraemia, extreme precipitation	4,804	5,900,000	Multivariate binomial regression	IRR =1.03 (95% CI: 1.01, 1.05) at 1 day EPT_90_
9	Colston et al. 2020 [53]	Peru	2011–2012	Monthly lab–confirmed cases and floods	1,386	n/a	Interrupted time series	RR = 1.41 (95% CI: 1.01, 1.07)

a r = correlation coefficient, B = beta coefficient, RR = relative risk, OR = odds ratio, IRR = incidence rate ratio, n/a = not available, PR = precipitation range.

The studies by Bi et al. [22] in Australia and Carev et al. [23] in Croatia reported positive correlations. The studies used regression analyses and controlled for seasonality (using a categorical seasonal variable), lag effects, and long-term trends. Weekly maximum temperature had a positive impact on gastroenteritis in Brisbane but not in Adelaide in the Australian study [22]. Neither these studies nor another study in Denmark [24] found any association between Campylobacter gastroenteritis and precipitation. A study by Kuhn et al. [11] in Nordic countries that studied 64,034 cases over 15 years included cases of bacteraemia and reported a r of 0.09.

Two studies reported OR to show the positive association between the climate variables and gastroenteritis – an international study [24] (weekly maximum temperature) and another case-crossover study on outbreaks in England (daily total rainfall) [25] reported OR of 1.3 (95% CI: 1.08, 1.55) and 2.88 (95% CI: 0.29, 28.1), respectively. The time-series study by Fleury et al. [26] in two provinces in Canada reported a 2.2% increase in gastroenteritis in Alberta and 4.5% in Newfoundland–Labrador, respectively, per degree rise in weekly mean temperature. A study in Maryland, USA [16], analysed the association with extreme heat and precipitation and found an IRR of 1.04 (95% CI: 1.01, 1.08) and 1.03 (95% CI: 1.01, 1.05), respectively. Importantly, this study included cases of bacteraemia and found that higher La Niña periods have a greater impact on the incidence of infections compared to El Niño periods (IRR = 1.09).

#### Salmonella species

**Figure 4.**
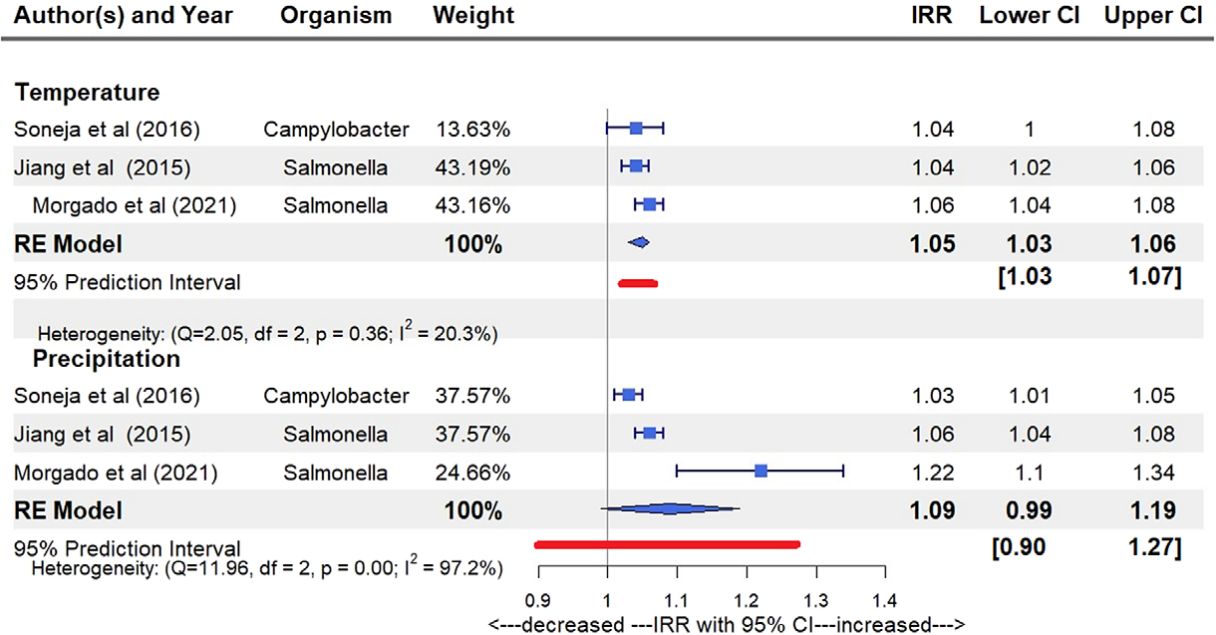
Pooled studies including bacteraemia climate estimated risk IRR. Pooled IRR indicating the health impacts associated with one unit increase in exceedance days for extreme temperature threshold 95th percentile (ETT_95_) and extreme precipitation threshold 90th percentile (EPT_90_), with 95% CIs.

The majority of typhoid and non-typhoidal salmonella infections are found in Africa and Asia. Salmonellosis is the most common cause of bacteraemia in African children [27]. This pathogen also contributes to significant DALY in developed countries (Figure 2) [27]. Studies on Salmonellosis were conducted in North America, Asia, Europe, and Australia. All studies on *Salmonella* with increases in monthly, weekly, daily, and extreme temperatures showed an association with a rise in cases regardless of the outcome measure used (Tables 4 and 5, Figure 3b). However, precipitation had different effects in temperate and tropical regions of the world. Four out of the 16 studies (25%) did not find a positive association with precipitation. Both climate variables had a positive association with bacteraemia in the USA (Figure 4).

**Table 4.** Studies on *Salmonella* sp. with temperature as the climate variable, stratified by type of temperature measurement

Number	Study	Region	Time period	Infection studied	Cases number	Population number	Analytical method	Key findings and reported statistic^a^
***Monthly mean temperature***								
1	Kim et al. 2015	South Korea	2003–2012	Monthly outbreaks of gastroenteritis	n/a	n/a	Pearson correlation	r = 0.75 Incidence calculated by dividing pathogen–specific outbreaks by total food–borne outbreaks
2	Akil et al. 2014 [2]	USA	2002–2011	Monthly outbreaks, analysis reported with cases	n/a	n/a	Regression analysis and neural network modelling	r = 0.76 1^0^ F rise in temperature led to 4 new cases. TR 35–95 °F.
3	Mun 2020 [32]	USA	2009–2016	Monthly lab–confirmed outbreaks compared with outbreaks in restaurants	n/a	n/a	Linear regression analysis	B = 0.01 with lag 4 weeks
4	Britton et al. 2010 [82]	NZ	1965–2006	Monthly lab–confirmed cases	n/a	n/a	Negative binomial regression	IRR = 1.15 (95% CI: 1.07, 1.24),15% rise in cases per degree rise in monthly average temp
5	Cherrie et al. 2018 [28]	England	1989–2014	Monthly lab–confirmed cases	n/a	n/a	ARMA	r = 0.37, *Salmonella enteritidis* and *Salmonella typhimurium* strongest correlation at 4 weeks
6	Ravel et al. 2010 [9]	Ontario, Canada	2005–2008	Monthly lab–confirmed cases	216	500,000	Poisson regression	r = 0.04
7	Grjibovski et al. 2013 [83]	Arkhangelsk, Russia	1992–2008	Monthly lab–confirmed cases	4,585	348,000	Negative binomial regression	B = 2.04 at lag 4 weeks, 2.04% rise per degree rise in temperature TR −20–20 °C
8	Kendrovski et al. 2011 [30]	Macedonia	1998–2008	Monthly lab–confirmed cases	3,890	2,052,722	Pearson correlation	r = 0.51 at 4–week lag TR 4–24 °C
9	Wang et al. 2012 [31]	Guizhou, China	1984–2007	Monthly lab–confirmed cases	n/a	n/a	Spearman rank correlation and wavelet analysis	r = 0.55 at 4–week lag TR 1.8–25.8 °C
10	Zhang et al. 2010 [86]	Townsville,A ustralia	1990–2005	Monthly lab–confirmed cases	1,170	186,000	Spearman correlation	B = 0.04 with max temperature (TR 24–34 ° C) B = 0.06 with min temp. (0–25 ° C) at lag 4 weeks
***Weekly mean temperature***								
11	Aik et al. 2018 [39]	Singapore	2005–2015	Weekly lab–confirmed infections	11,324	5,500,000	Multivariable regression analysis	IRR = 1.06 (95% CI: 1.02, 1.11) 6.3% increase per degree after 3 weeks. TR 25.3–30.1
12	Lake et al. 2009 [35]	UK	1981–2006	Weekly lab–confirmed gastroenteritis			Regression analysis	RR = 1.05 (95% CI: 1.03, 1.08)
13	Dewan et al. 2013 [33]	Bangladesh	2005–2009	Weekly lab–confirmed cases	n/a	n/a	Spatial and time series	RR = 1.8 (95% CI: 1.2, 2.8) at 4 weeks, 14.2% rise with 1–degree rise in temp. TR 20–30^0^ C
14	Fleury et al. 2006 [26]	Alberta, Canada	1992–2000	Weekly lab–confirmed cases	6,282	2,696,826	Generalized linear and additive model	Max RR = 1.02 (95% CI: 1.01, 1.02) at lag 2 weeks. Positive association with temperature in Alberta but not Newfoundland TR 15–40^0^ C
15	Robinson et al. 2022 [34]	Melbourne, Australia	2000–2019	Weekly lab–confirmed cases	29,421	5,000,000	Quasi–Poisson generalized linear model	RR = 1.1 (95% CI: 1.05, 1.2) at lag 4 weeks TR 15–35^0^ C
16	Zhang et al. 2008 [85]	Adelaide, Australia	1990–2004	Weekly lab–confirmed cases	4,740	1,100,000	Spearman correlation	B = 0.04 at lag 1 week, increase in cases up to 4 weeks
17	Zhang et al. 2010 [86]	Brisbane, Australia	1990–2005	Weekly lab–confirmed cases	5,294	1,600,000	Spearman correlation	B = 0.09 with max temp. (15–35^0^ C) and B = 0.06 with min temperature (5–25^0^ C) at lag 2 weeks
18	Nili et al. 2021 [21]	Iran	2008–2018	Stool, blood weekly lab–confirmed cases	569	1,952,435	Negative binomial generalized linear model	IRR = 1.04 (95% CI: 1.02, 1.06)
***Daily, annual and extreme temperature***								
19	Wang et al. 2018 [38]	Hong Kong	2002–2011	Daily admissions, daily mean temperature	4,828	7,340,000	DLNM and GAM	RR =6.13 (95% CI: 3.52, 10.67) TR 15–30^0^ C
20	Milazzo et al. 2016 [40]	Adelaide, Australia	1990–2012	Daily lab–confirmed cases, daily maximum temperature	7,845	n/a	Time–series Poisson regression	IRR 1.034 in summer months in Adelaide at lag 2 weeks, risk varied with serotypes TR 10–40^0^ C
21	Simpson et al. 2019 [84]	NSW, Australia	2001–2015	Annual lab–confirmed cases, mean annual temperature	514	n/a	CAR	RR = 1.31 (95% CI: 1.01, 1.68), more with *S. wangata* compared to typhimurium
22	Jiang et al. 2015 [18]	Maryland, USA	2000–2012	Extreme temperature, blood, stool	9,529	5,980,000	Negative binomial GEE	IRR = 1.04 (95% CI: 1.01, 1.07) ETT_95_
23	Morgado et al. 2021 [19]	Connecticut, USA	2004–2014	Extreme temperature, blood, stool	32,951	n/a	Negative binomial GEE	IRR =1.06 (95% CI: 1.04, 1.09) ETT_95_
24	Iyer et al. 2021 [36]	Gujarat, India	1995–2017	Monthly lab–confirmed enteric fever cases, extreme temperature > 95^th^ percentile	29,236	10,400,000	Negative binomial generalized linear model	RR = 1.01 (95% CI: 0.98, 1.04) TR 15–35^0^ C

*Note:* TR: 0–35 °C.

a r = correlation coefficient, B = beta coefficient, RR = relative risk, OR = odds ratio, IRR = incidence rate ratio, n/a = not available, DLNM = distributed lag non-linear model, GAM = generalized additive model, CAR = conditional autoregressive model, GEE = generalized estimating equation.

**Table 5.** Studies on *Salmonella* sp. with precipitation as the climate variable, stratified by type of precipitation measurement

Number	Study	Region	Time period	Infection studied	Cases number	Population number	Analytical method	Key findings and reported statistic^a^
***Weekly total precipitation***								
1	Aik et al. 2018 [39]	Singapore	2005–2015	Weekly lab–confirmed infections	11,324	5,500,000	Multivariable regression analysis	IRR = 1.01 (95% CI: 1.02, 1.02) at lag 2 weeks PR 0–440 mm
2	Dewan et al. 2013 [33]	Bangladesh	2005–2009	Weekly lab–confirmed cases			Spatial and time series	RR = 1.5 (95% CI: 1.2, 2.2) at 3 weeks PR 50–200 mm
3	Liu et al. 2018 [37]	Hunan, China	2005–2012	Weekly lab–confirmed cases	1,682	n/a	DLNM	RR = 1.46 (95% CI: 1.10, 1.92) after lag 1 week PR 0–200 mm
4	Robinson et al. 2022 [34]	Melbourne, Australia	2000–2019	Weekly lab–confirmed cases	29,421	5,000,000	Quasi–Poisson generalized linear model	No association PR 0–40 mm
5	Zhang et al. 2010 [86]	Brisbane, Australia	1990–2005	Weekly lab–confirmed cases	5,294	1,600,000	Spearman correlation	B = 0.002 with lag 2 weeks
***Monthly total precipitation***								
6	Zhang et al. 2010 [86]	Townsville, Australia		Monthly lab–confirmed cases	1,170	186,000	Poisson regression	B = 0.0006 at lag 12 weeks
7	Akil et al. 2014 [2]	USA	2002–2011	Monthly outbreaks	n/a	n/a	Regression analysis and neural network modelling	No correlation
8	Mun 2020 [32]	2009–2016	USA	Monthly lab–confirmed outbreaks compared with outbreaks in restaurants	n/a	n/a	Linear regression analysis	B = −0.02 with lag 4 weeks
9	Ravel et al. 2010 [29]	Ontario, Canada	2005–2008	Monthly lab–confirmed cases	216	500,000	Poisson regression	No association
10	Grjibovski et al. 2013 [83]	Arkhangelsk, Russia	1992–2008	Monthly lab–confirmed cases	4,585	348,000	Negative binomial regression	Uncertain association PR 0–150 mm
11	Wang et al. 2012 [31]	Guizhou, China	1984–2007	Monthly lab–confirmed cases	n/a	n/a	Spearman rank correlation and wavelet analysis	r = 0.48 at 4–week lag PR 5–437 mm
***Extreme and daily precipitation***								
12	Jiang et al. 2015 [18]	Maryland, USA	2000–2012	Blood, stool, extreme precipitation	9,529	5,980,000	Negative binomial GEE	IRR = 1.06 (95% CI: 1.04, 1.08) EPT_90_
13	Morgado et al. 2021 [19]	Connecticut, USA	2004–2014	Blood, stool, extreme precipitation	32,951	n/a	Negative binomial GEE	IRR = 1.22 (95% CI: 1.10, 1.35) EPT_95_
14	Iyer et al. 2021 [36]	Gujarat, India	1995–2017	Monthly lab–confirmed enteric fever cases, extreme precipitation	29,236	10,400,000	Negative binomial generalized linear model	RR = 1.01 (95% CI: 0.97, 1.05) PR 250–450 mm
15	Wang et al. 2018 [38]	Hong Kong	2002–2011	Daily admissions, daily precipitation	4,828	7,340,000	DLNM and GAM	RR =1.34 (95% CI: 0.98, 1.84) PR 0–100 mm
16	Zhang et al. 2008 [85]	Adelaide, Australia	1990–2004	Weekly lab–confirmed cases, daily rainfall	4,740	1,100,000	Spearman correlation	r = −0.02 (95% CI: −0.04, −0.003)

a r = correlation coefficient, B = beta coefficient, RR = relative risk, OR = odds ratio, IRR = incidence rate ratio, n/a = not available, DLNM = distributed lag non-linear model, GEE = generalized estimating equation, GAM = generalized additive model, PR = precipitation range.

Four studies measured monthly average temperature and reported a positive correlation of Salmonella gastroenteritis with ambient temperature. Cherrie et al. [28] performed a time-series analysis in England reporting r = 0.37 for temperature. A surveillance study in Ontario, Canada, by Ravel et al. [29] found monthly cases peaking in the summer months, while there was no association with precipitation. Similar seasonality was noted in a study in Macedonia [30] with a rise of 5.2% incidence per month with maximum monthly mean temperature. Lastly, Wang et al. [31] found an r = 0.55 for monthly temperature and r = 0.48 for monthly precipitation in Guizhou, China. Studies by Akil et al. [2] and Mun et al. [32] reported a positive correlation with an outbreak of infections. However, the association was tested with an actual number of infections in the study period in the former study.

Using RR as an outcome measure and weekly mean temperature for exposure, four studies reported a positive association with salmonella gastroenteritis. Three of these were time-series analyses. The first [26] in Alberta, Canada, showed a log RR increase of 1.2%; the second [33], in Dhaka, Bangladesh, reported an increase of 14.2% with a 1° rise in temperature for typhoid cases; and the third [34] in Melbourne, Australia, estimated a twofold increase at 33 °C compared to average weekly temperature. Lastly, Lake et al. [35] reported a RR of 1.05 for *S. typhimurium* and *S. enteritidis* infections in England. In contrast to the temperate regions of the world, four studies in Asian countries reported a positive association with precipitation. Three of these reported a rise in typhoid cases with increased rainfall and floods [33, 36, 37]. The study by Wang et al. [38] reported a rise in Salmonella hospitalizations in Hong Kong, along with a rise in daily precipitation.

For bacteraemia, two studies in the USA that included positive blood culture cases reported a positive association with extreme temperature and precipitation events. Firstly, the study by Morgado et al. [19] reported an IRR of 1.06 (95% CI: 1.04, 1.09). Similarly, the study by Jiang et al. [18] in Maryland, USA, reported an IRR of 1.041 (95% CI: 1.013, 1.069). Another study using IRR was a time-series analysis in Singapore [39] that examined weekly temperature (1 °C rise) and precipitation (10 mm rise) and reported a 4.3% increase and 0.8% increase in gastroenteritis, respectively. Lastly, Milazzo et al. [40] found an increased risk of *Salmonella* cases varies with serotypes in Adelaide, and Britton et al. reported an IRR of 1.15 (95% CI: 1.07, 1.24) in New Zealand with a rise in monthly average temperature.

#### Shigella species

Studies on Shigellosis [41–48] were predominantly from China, and all nine studies on temperature showed a positive association (Figure 3c). Nine of ten studies (90%) on extreme precipitation events like floods showed a positive association (Tables 6 and 7). (Figure 3c). Most studies found a rise in the incidence of gastroenteritis between 10 and 30 ° C temperature range. All the included studies had gastroenteritis as the predominant clinical manifestation, and no studies specified bacteraemia as an outcome.

**Table 6.** Studies on *Shigella* sp. with temperature as the climate variable, stratified by type of temperature measurement

Number	Study	Region	Time period	Infection studied	Cases number	Population number	Analytical method	Key findings and reported statistics^a^
***Daily mean temperature***								
1	Ai et al. 2022 [41]	China	2010–2018	Daily lab–confirmed cases and maximum temperature	n/a	n/a	Distributed lag non–linear model	RR = 1.15 (95% CI: 1.04, 1.28) Hot nights more associated than hot days. Short lag period of up to 7 days in China. TR 19.7–28^0^ C
2	Cheng et al. 2017 [43]	Hefei, China	2006–2012	Daily lab–confirmed gastroenteritis	n/a	n/a	Distributed lag non–linear model	RR = 1.03 (95% CI: 1.02, 1.05) at lag 1 week. Acute effects due to short incubation period. Effect sizes varied in different provinces in China. TR 10–34^0^ C
3	Li et al. 2016 [45]	Hefei, China	2006–2012	Daily lab–confirmed gastroenteritis	6,511	76,100,000	Poisson generalized linear regression	RR = 1.01 (95% CI: 1.00, 1.01) at lag 6 days TR 15–30^0^ C
4	Liu et al. 2020 [52]	China	2014–2016	Daily clinical and lab–confirmed gastroenteritis	396,134	n/a	DLNM	RR = 1.02 (95% CI: 1.01, 1.02) at lag 2 weeks. TR 15–30^0^ C
5	Wang y et al. 2021 [46]	Jilin, China	2008–2018	Daily clinical and lab–confirmed gastroenteritis	26,971	26,907,300	DLNM	RR = 1.88 (95% CI: 1.51, 2.34). Positive association for temperature up to 26 degrees. Reinforced by humidity and precipitation
6	Wen et al. 2016 [47]	Hefei, China	2006–2012	Daily clinical and lab–confirmed gastroenteritis	5,544	7,611,000	DLNM	RR = 1.08 (95% CI: 1.03, 1.13) diurnal temperature range above 8 degrees increased childhood dysentery cases
***Monthly mean temperature***								
7	Zhang et al. 2007 [48]	Jinan, China (temperate) Baoan (subtropical)	1987–2000	Monthly lab–confirmed gastroenteritis and maximum temperature	60,905	4,300,000	SARIMA	B = 0.11. Lag 4 weeks. Both monthly max (15–35^0^ C) and min mean temperature (8–25 ° C) related to rise in cases. 1–deg. rise leads to 12% rise in cases in Jinan B = 0.16 in Baoan
8	Lee et al. 2017 [44]	Vietnam	1999–2013	Monthly gastroenteritis	596,343	90,700,000	Negative binomial regression	r = 0.65, IRR = 1.06 (95% CI: 1.04, 1.09)
9	Aminharati et al. 2018 [42]	Yazd, Iran	2012–2015	Total lab–confirmed cases	68	1,138,533	Poisson regression	IRR = 1.25 (1.08, 1.45)

a r = correlation coefficient, B = beta coefficient, RR = relative risk, OR = odds ratio, IRR = incidence rate ratio, n/a = not available, TR = temperature ranges.

**Table 7.** Studies on *Shigella* sp. with precipitation as the climate variable, stratified by type of precipitation measurement

Number	Study	Region	Time period	Infection studied	Cases number	Population number	Analytical method	Key findings and reported statistics^a^
***Floods***								
1	Gao et al. 2016 [87]	Anhui, China	2007	Clinical and lab–confirmed cases	1,148	61,200,000	Poisson regression	OR = 1.04 (95% CI: 0.97, 1.12)
2	Liu et al. 2016 [55]	Huaihua, China	2005–2011	Weekly lab–confirmed cases	3,709	4,740,000	DLNM	RR = 1.32 (95% CI: 1.12, 1.56) with lag 1 week
3	Xu et al. 2017 [49]	Dalian, China	2004–2010	Weekly lab–confirmed cases	18,976	6,690,000	Generalized additive mixed model	r = 0.182 at lag 2 weeks RR = 1.17 (95% CI: 1.03, 1.33)
4	Liu et al. 2017 [50]	Baise, China	2004–2012	Monthly lab–confirmed cases	9,255	3,780,000	Mixed generalized additive model	r = 0.34 at lag 4 week RR = 1.40 (95% CI: 1.16, 1.69) r = 0.58 at lag 2 weeks RR = 1.78 (95% CI: 1.61, 1.77)
5	Liu et al. 2017 [54]	Guangxi, China	2004–2010	Monthly lab–confirmed cases	78,794	46,026,600	Poisson regression with generalized additive model	r = 0.34, lag 4 weeks r = 0.58, lag 2 weeks
6	Colston 2020 [53]	2011–2012	Peru	Total lab–confirmed cases	606	n/a	Modified Poisson regression	RR = 2.86 (95% CI: 1.81, 4.52)
***Monthly and weekly total precipitation***								
7	Hines et al. 2018 [88]	Oregon, USA	2015–2016	Total lab–confirmed cases, total precipitation in a week	105	4,000,000	Poisson regression	RR = 1.18 (95% CI: 1.06, 1.33) at lag 1 week
8	Lee et al. 2017 [44]	Vietnam	1999–2013	Monthly gastroenteritis	596,343	90,700,000	Negative binomial regression	r = 0.17 IRR = 1.04 (95% CI: 1.01, 1.07)
9	Aminharati et al. 2018 [42]	Yazd, Iran	2012–2015	Total lab–confirmed cases	68	1,138,533	Poisson regression	Not associated
10	Na et al. 2016 [61]	South Korea	2001–2009	Clinical and lab–confirmed cases	n/a	n/a	Multivariate log–linear model	RR = 3.1 (95% CI: 1.21, 7.92) at lag 2 weeks Cumulative precipitation of 209 mm

a r = correlation coefficient, B = beta coefficient, RR = relative risk, OR = odds ratio, IRR = incidence rate ratio, n/a = not available.

Three studies reported a correlation (r). Lee et al. [44] reported r = 0.65 for monthly average temperature and r = 0.17 for monthly precipitation in their study in Kon Tum Province, Vietnam. Two other studies [49, 50] found a rise in gastroenteritis cases in China after a lag of 2 weeks. Other Chinese studies [41, 43, 45, 47, 51, 52] reported an RR rise in Shigellosis with a rise in daily temperature. Li et al. [45] noted that each degree rise led to an increase of 1.6%, and children aged 0–5 years were largely affected. Wang et al. [51] noted that ambient temperature was the most important factor regardless of the climate zone studied. Also, temperate cities in China were more affected than subtropical cities. Further, studies in China [49, 53–55] revealed a positive association between Shigellosis cases and floods, with an increased incidence for up to three weeks. The risk was increased with short-term and severe floods and reduced with flood duration.

#### Vibrio species – cholera and non-cholera strains

Cholera is a major public health burden in Africa and Asia (Figure 2), and a majority of the studies on cholera were conducted in these continents. All nine studies on temperature and seven out of eight studies on precipitation showed a positive association with gastroenteritis (Figure 3d). The temperature range of rise in cases was 15–40 ° C. The study by Ruiz-Moreno et al. [56] extensively investigated the rainfall–cholera relationship in Madras and explained the dual peak in annual cases by the differential effects of rainfall in endemic and epidemic areas. Generally, a complex relationship between rainfall and ambient temperature and cholera varies across regions (Table 8 and Table 9). The study by Ali et al. in matrix laboratory (MATLAB), Bangladesh, found that for an increase in sea surface temperature by 1 °C, there was a 25% increase in cholera incidence in the current month and a 6% increase in incidence with per degree Celsius rise in ambient temperature [57]. Two other studies reported a correlation of 0.204 for daily temperature [58] and 0.42 for monthly precipitation [59]. Only two studies reported the relationship between *Vibrio* infections and precipitation using RR as the measure of effect: one for cholera [60] and the other for non-Vibrio cholera infections [61]. The cholera study reported a RR of 1.05 (1.04, 1.06) at lag 6 weeks, and the study on *Vibrio Vulnificus* infections reported a RR of 5.06 (95% CI: 2.41, 10.64) at lag 2 weeks.

**Table 8.** Studies on *Vibrio* sp. with temperature as the climate variable, stratified by type of temperature measurement

Number	Study	Region	Time period	Infection studied	Cases number	Population number	Analytical method	Key findings and reported statistics^a^
***Monthly mean temperature***								
1	Kim et al. 2015	South Korea	2003–2012	Monthly mean temperature and outbreaks of *V. parahaemolyticus*	n/a	n/a	Correlation analysis	r = 0.69 Incidence calculated by dividing pathogen–specific outbreak by total food–borne outbreaks
2	Ali et al. 2013 [57]	Bangladesh	1988–2001	Monthly lab–confirmed gastroenteritis and cholera	4,157	210,000	SARIMA	B = 0.41, r = 0.04 at lag 4 weeks. Minimum temperature increases of one degree Celsius in the current month led to 6% increase in cases
3	Baker et al. 2013 [89]	Baltic countries	1982–2010	Monthly sea temperature and all *Vibrio* infections	280	n/a	ARIMA	RR = 1.93 at lag 52 weeks. Highest mortality with *Vibrio vulnificus* infections
4	Reyburn et al. 2011 [93]	Zanzibar	2002–2008	Monthly lab–confirmed cholera cases	3,245	1,100,000	SARIMA	B = 2.21 at lag 16 weeks. Temperature and rainfall interacted significantly at 1 month lag. 1–degree rise in temp led to twofold rise in cases at 4 months. TR 0–22^0^ C
***Daily and weekly temperature***								
5	Hsiao et al. 2016 [91]	Taiwan	2000–2011	Monthly lab–confirmed *V. parahaemolyticus*	3,870 outbreaks	n/a	ARIMA	r = 1. Average temperature, ocean temperature, and salinity had a significant impact but not rainfall. TR 15–30^0^ C
6	Islam et al. 2009 [92]	MATLAB, Bangladesh		cholera	n/a	n/a	Regression and principal component analysis	Synergistic effect of temperature and sunshine hrs TR 18–30^0^ C
7	Asadgol et al. 2019 [58]	Qom, Iran	1998–2016	Daily lab–confirmed cholera cases	1,243	1,000,000	Artificial neural network modelling and gamma test	r = 0.20 at lag 4 weeks. Warm and dry environments increased the incidence TR 18–40^0^ C
8	Davis et al. 2021 [62]	Washington	2013–2018	Annual lab–confirmed *V. parahaemolyticus* cases	112	n/a	Multivariate logistic regression	OR = 2.16 (95% CI: 1.15, 4.05) Regional variations in the association. Also studied oyster tissue temperature
9	Fernandez et al. 2009 [60]	Lusaka, Zambia	2003–2006	Weekly lab–confirmed cholera (Ogawa)	13,069	1,284,642	Poisson autoregressive	RR = 1.05 (95% CI: 1.04, 1.06) at lag 6 weeks. 1–deg. rise in temp explained 5.2% rise in cholera cases. Favours the growth of algae and copepods. TR 21–26^0^ C

a r = correlation coefficient, B = beta coefficient, RR = relative risk, OR = odds ratio, IRR = incidence rate ratio, n/a = not available, TR = temperature ranges.

**Table 9.** Studies on *Vibrio* sp. with precipitation as the climate variable, stratified by type of precipitation measurement

Number	Study	Region	Time period	Infection studied	Cases number	Population number	Analytical method	Key findings and reported statistics^a^
***Monthly total rainfall***								
1	de Magny et al. 2008 [90]	Kolkata, India	1998–2006	Monthly lab–confirmed cases	n/a	n/a	Wavelet analyses	r = 0.06 Surface runoff into rivers floods water supply increases algal bloom promoting *Vibrio*
2	Reyburn et al. 2011 [93]	Zanzibar	2002–2008	Monthly lab–confirmed cholera cases	3,245	1,100,000	SARIMA	B = 0.01 at lag 8 weeks. Temperature and rainfall interacted significantly at 1 month lag. PR 3–705 mm
3	Na et al. 2016 [61]	South Korea	2001–2009	Clinical and lab–confirmed cases	n/a	n/a	Multivariate log–linear model	RR = 5.06 (95% CI: 2.41, 10.64) at lag 2 weeks Cumulative precipitation of 209 mm
***Daily and weekly precipitation and floods***								
4	Eisenberg et al. 2013 [63]	Haiti	2010–2011	Lab–confirmed cases	4,662	n/a	DLNM	OR = 1.46 (95% CI: 1.32, 1.61) at lag 1 week PR 0–216 mm
5	Hsiao et al. 2016 [91]	Taiwan	2000–2011	Monthly lab–confirmed *V. parahaemolyticus*, daily maximum rainfall	3,870 outbreaks		ARIMA	R = 0. 56. Average temp, ocean temp, and salinity had a significant impact but not rainfall
6	Asadgol et al. 2019 [58]	Qom, Iran	1998–2016	Daily lab–confirmed cholera cases	1,243	1,000,000	Artificial neural network modelling and gamma test	r = −0.23 at lag 4 weeks. PR 9–35 mm
7	Fernandez et al. 2009 [60]	Lusaka, Zambia	2003–2006	Weekly lab–confirmed cholera (Ogawa), weekly total precipitation	13,069	1,284,642	Poisson autoregressive	RR = 1.02 (95% CI: 1.01, 1.04) at lag 3 weeks. PR 0–307 mm
8	Cash et al. 2014 [59]	Matlab, Bangladesh	1983–2010	Monthly lab–confirmed cases, floods	n/a	n/a	Pearson correlation	r = 0.42

a r = correlation coefficient, B = beta coefficient, RR = relative risk, OR = odds ratio, IRR = incidence rate ratio, n/a = not available, PR = precipitation range.

Non-cholera strains are predominantly associated with wound infections and septicaemia. These infections rise with sea surface temperature (Table 6). A case–control analysis for *V. parahaemolyticus* infections in Washington, USA, reported an OR of 2.16 (95% CI: 1.15, 4.05) with yearly temperature [62], while a modelling study in Haiti showed an OR of 1.46 (95% CI: 1.32, 1.16) for daily precipitation [63]. An observational German study by Brehm et al. [64] noted an association between heat waves and increased *Vibrio* cases. Of 63 cases, 38 with wound infections and one with septicaemia were found in cases who had recreational exposure to the Baltic Sea or consumed shrimp from the sea after heatwave events.

#### Listeria species

Only one study by Chersich et al. [65] addressed the possibility of climate factors and Listeriosis. This discussed an outbreak of invasive Listeriosis in South Africa that resulted in 180 deaths. The source was traced to a food production facility that processed ‘ready-to-eat’ meat products. The risks identified were the impacts of temperature augmenting replication cycles of the bacterium, hot climate leading to breakdown in the food cooling chain, and the increased use of contaminated surface water.

#### Pooled estimates for bacteraemia

Four studies reported IRR for cases that included bacteraemia with ambient temperature and precipitation as climate variables [16, 18–20]. Our meta-analysis combined three of these studies as they included extreme heat and precipitation as exposure variables (Figure 4). These studies used extreme temperature threshold 95% percentile (ETT_95_) and extreme precipitation threshold (EPT_90_) and showed a pooled IRR of 1.05 (95% CI: 1.03, 1.06) associated with a unit increase in ETT_95_ exceedance days and 1.09 (95% CI: 0.99, 1.19) associated with a unit increase in EPT_90_ exceedance days (Figure 4).

## Discussion

In this systematic review and meta-analysis, we conducted a comprehensive synthesis of the impact of ambient temperature and precipitation on five food-borne pathogens based on published data between 2001 and 2021. In Europe, Australia, and North America, where Campylobacteriosis is predominant, a positive association was found with a rise in ambient temperature. Similarly, Salmonellosis incidence rose worldwide with temperature, with all studies showing a positive association. In contrast, the association with precipitation for both pathogens was less evident in temperate regions of the world. Shigellosis and Vibrio infections, more predominant in Africa and Asia, had a positive association with both temperature and excess precipitation. The positive association between these climate variables and illness was also consistent among studies where bacteraemia cases were included.

The findings of our review and meta-analysis are consistent with prior reviews on ambient temperature rise and infections from Campylobacter, Salmonella, and Shigella species [66, 67]. The majority of the studies for Campylobacter and Salmonella were either of high or moderate quality, which increased the reliability of the outcome measures, particularly for the two pathogens. This is the first review to demonstrate a positive association for studies including bacteraemia from Campylobacter and Salmonella species as an outcome.

The variable effect of climate variables on bacterial food pathogens in different regions of the world needs an understanding of not only the pathogen’s multiplication risks but also the modes of transmission and human behavioural factors. Campylobacter studies mostly found a lag period of 4–5 weeks, suggesting food contamination as the likely reason for the rise in incidence. The increase in cases in summer, particularly in Europe, seems to be related to the changes in behaviour among the people, for example, having more barbecues, outdoor parties, and contact with infected animals. The rise in temperature also increases the risk of infection in broiler flocks, and any errors in the cold chain of food transport can increase the risk in humans [4]. With projected rises in ambient temperature, Campylobacter infection seasonality will be longer and not restricted just to summer months. This could translate to an increase of infections by 200% in the Nordic countries by the end of the century [3]. Although Campylobacter sp. replicates in humid conditions, a positive association with precipitation has not been consistently found. Possible explanations are, firstly, a paucity of studies and, secondly, heterogeneity in using the time of exposure of precipitation. A significant impact may not be found when daily total precipitation is averaged out to weekly estimates. Studies using excessive precipitation in a day showed a significant association [16, 25]. Although the studies on Campylobacter bacteraemia do not mention the incidence of bacteraemia separately, the proportion of bacteraemia would also be expected to rise with current climate trends.

*Salmonella* replication is enhanced with the rise in temperature, which explains the cyclical rise in cases in late summer in temperate regions of the world. The variation in temperature in equatorial regions is less pronounced, which could explain the lesser impact of temperature in these areas [10]. However, seasonal monsoons in these regions lead to a rise in enteric fever every year, as flooding is a risk for transmission of enteric fever [10]. This can explain the positive association with lagged effects in Asian countries. In contrast, only excessive daily precipitation positively affected temperate regions of the world. The review by Saad [10] includes 16 datasets with *Salmonella* bacteraemia and showed a positive association with temperature and rainfall. An increase in ambient temperature over the coming years would significantly impact the incidence of salmonellosis worldwide, particularly in the non-equatorial regions, which would also translate to an increase in hospitalizations.

Bacillary dysentery cases from *Shigella* species also rise with temperature as the bacterium replicates more and food-borne transmission rises. The difference compared to the other bacterial food pathogens is the short lag period, as the incubation period is short. With precipitation, the most consistent association is with floods. This is more obvious in low socio-economic areas in China, where poor access to clean drinking water during floods increases the risk of transmission [51]. Given that most cases are diagnosed with stool specimens, our review found no studies specific to bacteraemia. Only three studies were of high quality, as most other studies had biases with confounding and exposure.

Ambient temperature promotes *Vibrio* species growth, and increases in algal blooms contribute as well [68]. For *non-cholera* species, water temperature and salinity are the two most important risk factors for growth. With the warming of the oceans, coastal regions will face increased sepsis cases from these species [69]. Heat waves have helped spread *Vibrio sp.* to higher latitudes, and a mini-review predicted that infections might quadruple in the coming years [70]. Cholera cases in Africa and India have a complex relationship with climate variables. In the dry season, the rise in cases is chiefly due to increased ambient temperature. During the monsoon, the dilutional effect of rainfall on water salinity leads to a reduction in the number of cases. After a lag period, due to increased contact with contaminated water, there is another peak of cases [56]. A study by Koelle et al. [71] in MATLAB, Bangladesh, demonstrated an association of outbreaks with monsoons and a lag period as long as eight months. The authors also noted that if herd immunity is high after a recent outbreak, climate variables had a limited impact on cholera transmission. Four studies were of high quality, while the rest had confounding and selection biases. In the future, the prediction is that *Vibrio* infections will rise and expand geographically with current climate trends [69]. This would include cases of bacteraemia and lead to high mortality.

We acknowledge the following limitations in this review and meta-analysis. First, some studies conducted in non-English languages were excluded. Second, the absence of data on the proportion of cases of bacteraemia in the studies prevented an accurate prediction of the genuine impact of climate on this severe outcome. Thirdly, only one author did the screening, and only MEDLINE was updated in March 2023 to capture any missing recent studies. The maximum number of publications was obtained from the databases, and the potential for missed publications, although possible, is low. Many studies reported outcomes for multiple pathogens but reported outcome measures separately, reducing the chance of any reporting bias. Lastly, although we developed our study protocol a priori (available on request from the corresponding author upon request), time constraints prevented us from registration or publication before this was completed. Despite these acknowledged limitations, the findings in this study are important and valid.

In summary, this is the first review that provides a comprehensive overview of the complex interactions between the intensity and timing of climate variable exposure and the incidence of pathogen-specific infections. Studies that included cases of *Campylobacter* and *Salmonella* bacteraemia reported a rise in incidence with ambient temperature and precipitation. Further research is needed to study the impact of a surge in food pathogen bacteraemia with current trends in climate change.

## Supporting information

Manchal et al. supplementary material Manchal et al. supplementary material

## Data Availability

All relevant data are presented in the manuscript.

## Supplementary material

For supplementary material accompanying this paper visit http://doi.org/10.1017/S0950268824000839. click here to view supplementary material
