# A systematic review and meta-analysis of ambient temperature and precipitation with infections from five food-borne bacterial pathogens – CORRIGENDUM

**DOI:** 10.1017/S0950268824001742

**Published:** 2024-12-10

**Authors:** Naveen Manchal, Megan K. Young, Maria Eugenia Castellanos, Peter Leggat, Oyelola Adegboye

**Affiliations:** 1 Public Health and Tropical Medicine, College of Public Health, Medical and Veterinary Sciences, James Cook University, Townsville, QLD 4811, Australia; 2 Metro North Public Health Unit, Metro North Hospital and Health Service, Brisbane, Australia; 3 School of Medicine and Dentistry, Griffith University; 4 School of Public Health, University of Queensland; 5 Australian Institute of Tropical Health and Medicine, James Cook University, Townsville, QLD 4811, Australia; 6 World Health Organization Collaborating Centre for Vector-Borne and Neglected Tropical Diseases, James Cook University, Townsville, QLD 4811, Australia; 7 School of Public Health, Faculty of Health Sciences, University of the Witwatersrand, Johannesburg, South Africa; 8 Menzies School of Health Research, Charles Darwin University, Darwin, NT 0810, Australia

## Error in Figure/Table

In the published article, there was an error in Table 2a as published. Instead of r=0.01 and B=0.01 reported statistic for Bi 2008, it should be r=0.009 at lag 6 weeks and B=0.007 at lag 9 weeks. Also, instead of B=7.32 reported statistic for Djennad 2019, it should be 7.32 e-0.03.

The authors apologize for this error and state that this does not change the scientific conclusions of the article in any way.

In the published article there was an error in Table 2b. The key reported statistic for Djennad 2019 was B=9.36. The corrected statistic should be B=-2.439 e-03

In the published article, there was an error in Table 4b. The reported key reported statistic for Liu et al 2017 was r=0.34, lag 4 weeks r=0.58, lag 2 weeks. The corrected statistic should be r=0.40, lag 4 weeks and r=0.56, lag 0 week.

In the published article, there was an error in Table 1. Regarding the rise in precipitation, “6 out of 9 studies reported positive association”. The corrected statement is “5 out of 9 studies reported positive association”.

In the published article, there was an error in Figure 3A. The corrected figure is below

**Figure 3.**
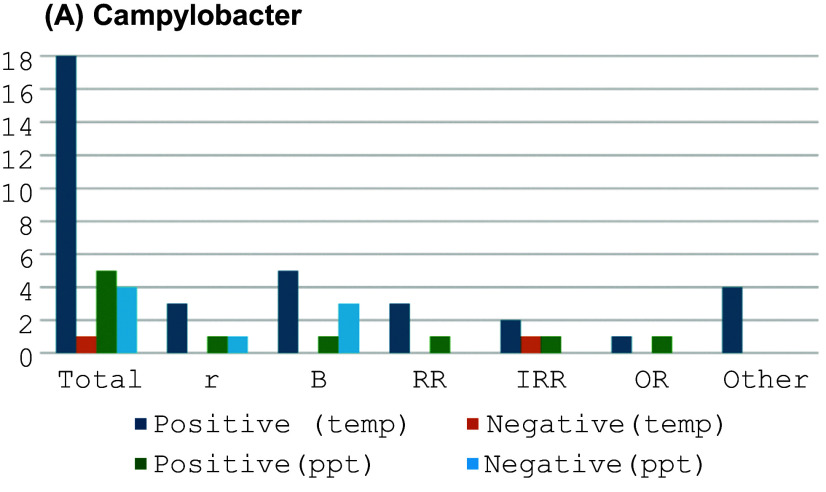
Graphs summarizing the estimated effects (r, beta, RR, IRR, and OR) of temperature and precipitation on specific pathogens. (a) Campylobacter.

The authors apologize for this error and state that this does not change the scientific conclusions of the article in any way.

## Text Correction

In the published article, there was an error.

A correction has been made to section “*3.2.1 Campylobacter species”*

This sentence previously stated: “With precipitation, six out of nine studies described a positive association (Figure 3A).”

The corrected sentence appears below:

“With precipitation, five out of nine studies described a positive association (Figure 3A).”

The authors apologize for this error and state that this does not change the scientific conclusions of the article in any way.
