# Supplementary material for: A systematic review and meta-analysis of ambient temperature and precipitation with infections from five food-borne bacterial pathogens
Source: Epidemiol Infect. 2024 Aug 22;152:e98. doi: 10.1017/S0950268824000839 (PMC11736460; doi:10.1017/S0950268824000839)
Supplement: Manchal et al. supplementary material [file S0950268824000839sup001.docx]

**Supplementary material**

Search parameters

Search parameters for PubMed and Scopus databases were “climate variables and *Salmonella* infections”, “climate change and *Shigella* infections”, “Precipitation and *Campylobacter* infections”, “climate change and *Listeria* infections” and “Climate change and *Vibrio* infections”. Filters included were “human” and “English”. The last search date for these databases was 7 July 2022. Web of Science search strategy included TS=climate AND (each organism) and English. The last date for this search was 29 July 2022. The search strategy for MEDLINE(Ovid) included two combined search terms: **Salmonella*/ or *Shigella*.mp. or *Campylobacter*.mp. or *Vibrio*.mp. or *Listeria*.mp AND *climate/ or temperature.mp. or precipitation.mp. or heatwaves.mp.  Only articles in English and on humans were included. The last date for the search for this database was 9 Ma

SUPPLEMENTARY MATERIAL

**Table S1- Qualitative analysis of studies in review using the ROBINS E-tool.**

| Study | Type of study | Risk of bias from confounding | Bias from missing data | Exposure bias | Selection bias | Outcome and reporting bias | Quality |
| --- | --- | --- | --- | --- | --- | --- | --- |
| 1. Auld 2004[45] | epidemiological |  |  |  |  |  | low |
| 1. Bi 2008[8] | Ecological |  |  |  |  |  | high |
| 1. Carev 2018[9] | surveillance |  |  |  |  |  | high |
| 1. Colston 2020[46] | ITS |  |  |  |  |  | moderate |
| 1. Djennad 2019[47] | Time series |  |  |  |  |  | moderate |
| 1. Kovats 2005[82] | Surveillance |  |  |  |  |  | high |
| 1. Kuhn 2020[2] | Surveillance |  |  |  |  |  | moderate |
| 1. Lake 2019[48] | Surveillance |  |  |  |  |  | high |
| 1. Nichols 2009[10] | Case crossover |  |  |  |  |  | moderate |
| 1. Onozuka and Hashizume 2011[49] | Surveillance |  |  |  |  |  | high |
| 1. Park 2018[50] | Epidemiologic |  |  |  |  |  | moderate |
| 1. Patrick 2004[51] | Epidemiologic |  |  |  |  |  | moderate |
| 1. Rosenberg 2018[52] | Surveillance |  |  |  |  |  | high |
| 1. Sanderson 2018[53] | Epidemiologic |  |  |  |  |  | moderate |
| 1. Soneja 2016[3] | Surveillance |  |  |  |  |  | moderate |
| 1. Thomas 2006[54] | Case crossover |  |  |  |  |  | moderate |
| 1. Vuckovic 2011[55] | Epidemiologic |  |  |  |  |  | High |
| 1. Weisent 2014[4] | Surveillance |  |  |  |  |  | high |
| 1. Tam 2005 [56] | Time series |  |  |  |  |  | High |
| 1. Milazzo 2017[57] | Time series |  |  |  |  |  | High |
| 1. Spencer et al 2012[58] | Time series |  |  |  |  |  | Moderate |
| 1. White et al 2009 [59] | Time series |  |  |  |  |  | Low |
| 1. Yun et al 2016[60] | Time series |  |  |  |  |  | Moderate |
| 1. Aik 2018[22] | Time series |  |  |  |  |  | moderate |
| 1. Akil 2014[1] | Time series |  |  |  |  |  | high |
| 1. Britton 2010[61] | epidemiologic |  |  |  |  |  | moderate |
| 1. Cherrie 2018[12] | Surveillance |  |  |  |  |  | moderate |
| 1. Dewan 2013[17] | Time series |  |  |  |  |  | moderate |
| 1. Fleury 2006[11] | Time series |  |  |  |  |  | moderate |
| 1. Monter et al[62] | Modelling |  |  |  |  |  | low |
| 1. Grjibovski 2013[63 | Time series |  |  |  |  |  | high |
| 1. Iyer 2021[19] | epidemiologic |  |  |  |  |  | moderate |
| 1. Jiang 2015[5] | epidemiologic |  |  |  |  |  | high |
| 1. Kelly hope 2008[64] | epidemiologic |  |  |  |  |  | moderate |
| 1. Kendrovski 2011[14] | epidemiologic |  |  |  |  |  | high |
| 1. Liu 2018[20] | epidemiologic |  |  |  |  |  | high |
| 1. Milazzo et al 2016[23] | epidemiologic |  |  |  |  |  | high |
| 1. Morgado et al 2021[6] | epidemiologic |  |  |  |  |  | high |
| 1. Mun 2020[16] | epidemiologic |  |  |  |  |  | high |
| 1. Nili 2021[7] | ecological |  |  |  |  |  | moderate |
| 1. Ravel et al[13] | epidemiologic |  |  |  |  |  | moderate |
| 1. Robinson et al[18] | Time series |  |  |  |  |  | moderate |
| 1. Simpson et al 2019[65] | ecological |  |  |  |  |  | high |
| 1. Wang et al 2012[15] | epidemiologic |  |  |  |  |  | moderate |
| 1. Wang et al 2018[21] | Time series |  |  |  |  |  | high |
| 1. Zhang et al 2008[66] | surveillance |  |  |  |  |  | high |
| 1. Zhang et al 2010[67] | epidemiologic |  |  |  |  |  | high |
| 1. Ai et al 2022[24] | Modelling |  |  |  |  |  | moderate |
| 1. Cheng et al 2017[25] | Modelling |  |  |  |  |  | high |
| 1. Gao et al2016[68] | modelling |  |  |  |  |  | high |
| 1. Hines et al 2018[69] | Time series |  |  |  |  |  | moderate |
| 1. Lee et al 2017[26] | Ecological study |  |  |  |  |  | moderate |
| 1. Li et al 2016[27] | Modelling study |  |  |  |  |  | moderate |
| 1. Liu et al 2017[83] | Modelling study |  |  |  |  |  | low |
| 1. Liu et al 2016[34] | Modelling study |  |  |  |  |  | moderate |
| 1. Liu et al 2017[31] | Modelling study |  |  |  |  |  | low |
| 1. Liu et al 2020[33] | epidemiologic |  |  |  |  |  | moderate |
| 1. Na et al 2016[40] | Modelling study |  |  |  |  |  | moderate |
| 1. Naumova et al 2007[70] | Modelling study |  |  |  |  |  | high |
| 1. Wang et al 2021[15] | Modelling study |  |  |  |  |  | low |
| 1. Wang y et al 2021[28] | Modelling study |  |  |  |  |  | moderate |
| 1. Wen et al 2016[29] | Modelling study |  |  |  |  |  | moderate |
| 1. Zhang et al 2007[30] | Modelling study |  |  |  |  |  | moderate |
| 1. Zhang et al 2017[44] | Modelling study |  |  |  |  |  | low |
| 1. Ali et al 2013[36] | Time series study |  |  |  |  |  | moderate |
| 1. Asadgol et al 2019[37] | Modelling study |  |  |  |  |  | high |
| 1. Baker et al 2013[71] | Modelling study |  |  |  |  |  | high |
| 1. Barachhini et al 2017[72] | Modelling study |  |  |  |  |  | low |
| 1. Bouma and Pascual 2001[73] | Modelling study |  |  |  |  |  | low |
| 1. Brehm et al 2021[43] | Retrospective cohort study |  |  |  |  |  | low |
| 1. Cash et al 2014[38] | Modelling study |  |  |  |  |  | high |
| 1. Davis et al 2021[41] | Case control study |  |  |  |  |  | moderate |
| 1. de Magny et al 2007[74] | Modelling study |  |  |  |  |  | low |
| 1. de Magny et al 2008[75] | Modelling study |  |  |  |  |  | moderate |
| 1. Eisenberg et al 2013[42] | Modelling study |  |  |  |  |  | high |
| 1. Fernandez et al 2009[39] | Time series study |  |  |  |  |  | moderate |
| 1. Hashizume et al 2010[76] | Modelling study |  |  |  |  |  | moderate |
| 1. Hashizume et al 2008[77] | Time series study |  |  |  |  |  | moderate |
| 1. Hsiao et al 2016[78] | Modelling study |  |  |  |  |  | moderate |
| 1. Islam et al 2009[79] | Modelling study |  |  |  |  |  | low |
| 1. Jutla et al 2015[80] | Modelling study |  |  |  |  |  | low |
| 1. Reyburn et al 2011[81] | Modelling study |  |  |  |  |  | moderate |
| 1. Ruiz-Moreno et al 2007[35] | Modelling study |  |  |  |  |  | low |

*Epidemiologic studies are retrospective observational studies using available case and climate data

**References**

[1] Akil L, Ahmad HA and Reddy RS (2014) Effects of climate change on salmonella infections. *Foodborne Pathogens and Disease* **11**(12), 974–980.

[2] Kuhn KG, et al. (2020) Campylobacteriosis in the Nordic countries from 2000 to 2015: Trends in time and space. *Scandinavian Journal of Public Health* **48**(8), 862–869.

[3] Soneja S, et al. (2016) Extreme precipitation events and increased risk of campylobacteriosis in Maryland, U.S.A. *Environmental Research* **149**, 216–221.

[4] Weisent J, et al. (2014) The importance of climatic factors and outliers in predicting regional monthly campylobacteriosis risk in Georgia, USA. *International Journal of Biometeorology* **58**(9), 1865–1878.

[5] Jiang CS, et al. (2015) Climate change, extreme events and increased risk of salmonellosis in Maryland, USA: Evidence for coastal vulnerability. *Environment International* **83**, 58–62.

[6] Morgado ME, et al. (2021) Climate change, extreme events, and increased risk of salmonellosis: Foodborne diseases active surveillance network (FoodNet), 2004–2014. *Environmental Health* **20**(1), 105.

[7] Nili S, et al. (2021) The effect of meteorological variables on salmonellosis incidence in Kermanshah, west of Iran: A generalized linear model with negative binomial approach. *Journal of Environmental Health Science and Engineering* **19**(1), 1171–1177.

[8] Bi P, et al. (2008) Weather and notified campylobacter infections in temperate and sub-tropical regions of Australia: An ecological study. *Journal of Infection* **57**(4), 317–323.

[9] Carev M, Tonkić M and Boban N (2018) A six-year epidemiological surveillance study in Split-Dalmatia County, Croatia: Urban versus rural differences in human campylobacteriosis incidence. *International Journal of Environmental Health Research* **28**(4), 407–418.

[10] Nichols G, et al. (2009) Rainfall and outbreaks of drinking water related disease and in England and Wales. *Journal of Water and Health* **7**(1), 1–8.

[11] Fleury M, et al. (2006) A time series analysis of the relationship of ambient temperature and common bacterial enteric infections in two Canadian provinces. *International Journal of Biometeorology* **50**(6), 385–391.

[12] Cherrie MPC, et al. (2018) Pathogen seasonality and links with weather in England and Wales: A big data time series analysis. *BMC Public Health* **18**(1), 1067.

[13] Ravel A, et al. (2010) Seasonality in human salmonellosis: Assessment of human activities and chicken contamination as driving factors. *Foodborne Pathogens and Disease* **7**(7), 785–794.

[14] Kendrovski V, Karadzovski Z and Spasenovska M (2011) Ambient maximum temperature as a function of salmonella food poisoning cases in the republic of Macedonia. *North American Journal of Medical Sciences* **3**(6), 264–267.

[15] Wang LX, et al. (2012) Association between the incidence of typhoid and paratyphoid fever and meteorological variables in Guizhou, China. *Chinese Medical Journal (Engl)* **125**(3), 455–460.

[16] Mun SG (2020) The effects of ambient temperature changes on foodborne illness outbreaks associated with the restaurant industry. *International Journal of Hospitality Management* **85**, 102432.

[17] Dewan AM, et al. (2013) Typhoid fever and its association with environmental factors in the Dhaka metropolitan area of Bangladesh: A spatial and time-series approach. *PLoS NeglectedTropical Diseases* **7**(1), e1998.

[18] Robinson EJ, et al. (2022) Effect of temperature and rainfall on sporadic salmonellosis notifications in Melbourne, Australia 2000–2019: A time-series analysis. *Foodborne Pathogens and Disease* **19**, 341–348.

[19] Iyer V, et al. (2021) Role of extreme weather events and El Niño southern oscillation on incidence of enteric fever in Ahmedabad and Surat, Gujarat, India. *Environmental Research* **196**, 110417.

[20] Liu Z, et al. (2018) Association between floods and typhoid fever in Yongzhou, China: Effects and vulnerable groups. *Environmental Research* **167**, 718–724.

[21] Wang P, Goggins WB and Chan EYY (2018) Associations of salmonella hospitalizations with ambient temperature, humidity and rainfall in Hong Kong. *Environment International* **120**, 223–230.

[22] Aik J, et al. (2018) Climate variability and salmonellosis in Singapore - A time series analysis. *Science of the Total Environment* **639**, 1261–1267.

[23] Milazzo A, et al. (2016) The effect of temperature on different salmonella serotypes during warm seasons in a Mediterranean climate city, Adelaide, Australia. *Epidemiology and Infection* **144**(6), 1231–1240.

[24] Ai S, et al. (2022) Effect and attributable burden of hot extremes on bacillary dysentery in 31 Chinese provincial capital cities. *Science of the Total Environment* **832**, 155028.

[25] Cheng J, et al. (2017) Impacts of ambient temperature on the burden of bacillary dysentery in urban and rural Hefei, China. *Epidemiology and Infection* **145**(8), 1567–1576.

[26] Lee HS, et al. (2017) Seasonal and geographical distribution of bacillary dysentery (shigellosis) and associated climate risk factors in Kon Tum Province in Vietnam from 1999 to 2013. *Infectious Diseases of Poverty* **6**, 113.

[27] Li K, et al. (2016) Daily temperature change in relation to the risk of childhood bacillary dysentery among different age groups and sexes in a temperate city in China. *Public Health* **131**, 20–26.

[28] Wang Y, et al. (2021) Effect of temperature and its interaction with other meteorological factors on bacillary dysentery in Jilin Province, China. *Epidemiology and Infection* **149**, e121.

[29] Wen LY, et al. (2016) The association between diurnal temperature range and childhood bacillary dysentery. *International Journal of Biometeorology* **60**(2), 269–276.

[30] Zhang Y, et al. (2007) Climate variations and bacillary dysentery in northern and southern cities of China. *Journal of Infection* **55**(2), 194–200.

[31] Liu X, et al. (2017) The effects of floods on the incidence of bacillary dysentery in Baise (Guangxi Province, China) from 2004 to 2012. *International Journal of Environmental Research and Public Health* **14**(2), 179.

[32] Wang S, et al. (2021) Real-time forecasting and early warning of bacillary dysentery activity in four meteorological and geographic divisions in China. *Science of the Total Environment* **761**, 144093.

[33] Liu Z, et al. (2020) Daily temperature and bacillary dysentery: Estimated effects, attributable risks, and future disease burden in 316 Chinese cities. *Environmental Health Perspectives* **128**(5), 57008.

[34] Liu X, et al. (2016) Quantitative analysis of burden of bacillary dysentery associated with floods in Hunan, China. *Science of the Total Environment* **547**, 190–196.

[35] Ruiz-Moreno D, et al. (2007) Cholera seasonality in Madras (1901–4940): Dual role for rainfall in endemic and epidemic regions. *EcoHealth* **4**(1), 52–62.

[36] Ali M, et al. (2013) Time series analysis of cholera in Matlab, Bangladesh, during 1988–2001. *Journal of Health Population and Nutrition* **31**(1), 11–19.

[37] Asadgol Z, et al. (2019) The effect of climate change on cholera disease: The road ahead using artificial neural network. *PLoS One* **14**(11), e0224813.

[38] Cash BA, et al. (2014) Cholera and shigellosis: Different epidemiology but similar responses to climate variability. *PLoS One* **9**(9), e107223.

[39] Fernandez MAL, et al. (2009) Influence of temperature and rainfall on the evolution of cholera epidemics in Lusaka, Zambia, 2003–2006: Analysis of a time series. *Transactions of the Royal Society of Tropical Medicine and Hygiene* **103**(2), 137–143.

[40] Na W, et al. (2016) Incidences of waterborne and foodborne diseases after Meteorologic disasters in South Korea. *Annals of Global Health* **82**(5), 848–857.

[41] Davis BJK, et al. (2021) A case-control analysis of traceback investigations for Vibrio parahaemolyticus infections (vibriosis) and pre-harvest environmental conditions in Washington state, 2013–2018. *Science of the Total Environment* **752**, 141650.

[42] Eisenberg MC, et al. (2013) Examining rainfall and cholera dynamics in Haiti using statistical and dynamic modeling approaches. *Epidemics* **5**(4), 197–207.

[43] Brehm TT, et al. (2021) Heatwave-associated vibrio infections in Germany, 2018 and 2019. *Eurosurveillance* **26**(41), 2002041.

[44] Zhang H, et al. (2017) Environmental drivers and predicted risk of bacillary dysentery in Southwest China. *International Journal of Environmental Research and Public Health* **14**(7), 782.

[45] Auld H, MacIver D and Klaassen J (2004) Heavy rainfall and waterborne disease outbreaks: The Walkerton example. *Journal of Toxicology and Environmental Health A* **67**(20–22), 1879–1887.

[46] Colston J, et al. (2020) Pathogen-specific impacts of the 2011–2012 La Nina-associated floods on enteric infections in the MAL-ED Peru cohort: A comparative interrupted time series analysis. *International Journal of Environmental Research and Public Health* **17**(2), 487.

[47] Djennad A, et al. (2019) Seasonality and the effects of weather on campylobacter infections. *BMC Infectious Diseases* **19**, 255.

[48] Lake IR, et al. (2019) Exploring campylobacter seasonality across Europe using the European surveillance system (TESSy), 2008 to 2016. *Euro Surveillance* **24**(13), 1800028.

[49] Onozuka D and Hashizume M (2011) Weather variability and paediatric infectious gastroenteritis. *Epidemiology and Infection* **139**(9), 1369–1378.

[50] Park MS, Park KH and Bahk GJ (2018) Combined influence of multiple climatic factors on the incidence of bacterial foodborne diseases. *Science of the Total Environment* **610–611**, 10–16.

[51] Patrick ME, et al. (2004) Effects of climate on incidence of campylobacter spp. in humans and prevalence in broiler flocks in Denmark. *Applied and Environmental Microbiology* **70**(12), 7474–7480.

[52] Rosenberg A, et al. (2018) Ambient temperature and age-related notified campylobacter infection in Israel: A 12-year time series study. *Environmental Research* **164**, 539–545.

[53] Sanderson RA, et al. (2018) Spatio-temporal models to determine association between campylobacter cases and environment. *International Journal of Epidemiology* **47**(1), 202–216.

[54] Thomas MK, et al. (2006) A role of high impact weather events in waterborne disease outbreaks in Canada, 1975–2001. *International Journal of Environmental Health Research* **16**(3), 167–180.

[55] Vucković D, et al. (2011) Epidemiologic characteristics of human campylobacteriosis in the county Primorsko-goranska (Croatia), 2003–2007. *Collegium Antropologicum* **35**(3), 847–853.

[56] Tam CC, et al. (2006) Temperature dependence of reported campylobacter infection in England, 1989–1999. *Epidemiology and Infection* **134**(1), 119–125.

[57] Milazzo A, et al. (2017) The effects of ambient temperature and heatwaves on daily campylobacter cases in Adelaide, Australia, 1990–2012. *Epidemiology and Infection* **145**(12), 2603–2610.

[58] Spencer SEF, et al. (2012) The spatial and temporal determinants of campylobacteriosis notifications in New Zealand, 2001–2007. *Epidemiology and Infection* **140**(9), 1663–1677.

[59] White ANJ, et al. (2009) Environmental determinants of campylobacteriosis risk in Philadelphia from 1994 to 2007. *EcoHealth* **6**(2), 200–208.

[60] Yun J, et al. (2016) Association between the ambient temperature and the occurrence of human salmonella and campylobacter infections. *Scientific Reports* **6**, 28442.

[61] Britton E, et al. (2010) Positive association between ambient temperature and salmonellosis notifications in New Zealand, 1965–2006. *Australian and New Zealand Journal of Public Health* **34**(2), 126–129.

[62] Flores Monter YM, et al. (2021) Edaphoclimatic seasonal trends and variations of the salmonella spp. Infection in Northwestern Mexico. *Infectious Disease Modelling* **6**, 805–819.

[63] Grjibovski AM, et al. (2013) Climate variations and salmonellosis in Northwest Russia: A time-series analysis. *Epidemiology and Infection* **141**(2), 269–276.

[64] Kelly-Hope LA, et al. (2008) Temporal trends and climatic factors associated with bacterial enteric diseases in Vietnam, 1991–2001. *Environmental Health Perspectives* **116**(1), 7–12.

[65] Simpson KMJ, et al. (2019) Divergent geography of salmonella Wangata and salmonella Typhimurium epidemiology in New South Wales, Australia. *One Health* **7**, 100092.

[66] Zhang Y, Bi P and Hiller J (2008) Climate variations and salmonellosis transmission in Adelaide, South Australia: A comparison between regression models. *International Journal of Biometeorology* **52**(3), 179–187.

[67] Zhang Y, Bi P and Hiller JE (2010) Climate variations and salmonella infection in Australian subtropical and tropical regions. *Science of the Total Environment* **408**(3), 524–530.

[68] Gao L, et al. (2016) Identifying flood-related infectious diseases in Anhui Province, China: A spatial and temporal analysis. *American Journal of Tropical Medicine and Hygeine* **94**(4), 741–749.

[69] Hines JZ, et al. (2018) Heavy precipitation as a risk factor for shigellosis among homeless persons during an outbreak - Oregon, 2015–2016. *Journal of Infection* **76**(3), 280–285.

[70] Naumova EN, et al. (2007) Seasonality in six enterically transmitted diseases and ambient temperature. *Epidemiology and Infection* **135**(2), 281–292.

[71] Baker-Austin C, et al. (2013) Emerging vibrio risk at high latitudes in response to ocean warming. *Nature Climate Change* **3**(1), 73–77.

[72] Baracchini T, et al. (2017) Seasonality in cholera dynamics: A rainfall-driven model explains the wide range of patterns in endemic areas. *Advances in Water Resources* **108**, 357–366.

[73] Bouma MJ and Pascual M (2001) Seasonal and interannual cycles of endemic cholera in Bengal 1891–1940 in relation to climate and geography. *Hydrobiologia* **460**, 147–156.

[74] de Magny GC, et al. (2007) Regional-scale climate-variability synchrony of cholera epidemics in West Africa. *BMC Infectious Diseases* **7**, 20.

[75] de Magny GC, et al. (2008) Environmental signatures associated with cholera epidemics. *Proceedings of the National Academy of Sciences of the United States of America* **105**(46), 17676–17681.

[76] Hashizume M, et al. (2010) Cholera in Bangladesh climatic components of seasonal variation. *Epidemiology* **21**(5), 706–710.

[77] Hashizume M, et al. (2008) The effect of rainfall on the incidence of cholera in Bangladesh. *Epidemiology* **19**(1), 103–110.

[78] Hsiao HI, Jan MS and Chi HJ (2016) Impacts of climatic variability on Vibrio parahaemolyticus outbreaks in Taiwan. *International Journal of Environmental Research and Public Health* **13**(2), 188.

[79] Islam MS, et al. (2009) Effects of local climate variability on transmission dynamics of cholera in Matlab, Bangladesh. *Transactions of the Royal Society of Tropical Medicine and Hygiene* **103**(11), 1165–1170.

[80] Jutla A, et al. (2015) Satellite based assessment of Hydroclimatic conditions related to cholera in Zimbabwe. *PLoS One* **10**(9), e0137828.

[81] Reyburn R, et al. (2011) Climate variability and the outbreaks of cholera in Zanzibar, East Africa: A time series analysis. *American Journal of Tropical Medicine and Hygiene* **84**(6), 862–869.

[82] Kovats RS, et al. (2005) Climate variability and campylobacter infection: An international study. *International Journal of Biometeorology* **49**(4), 207–214.

[83] Liu X, et al. (2017) Projected burden of disease for bacillary dysentery due to flood events in Guangxi, China. *Science of the Total Environment* **601–602**, 1298–1305.
